# Examination of Carbohydrate Products in Feces Reveals Potential Biomarkers Distinguishing Exclusive and Nonexclusive Breastfeeding Practices in Infants

**DOI:** 10.1093/jn/nxaa028

**Published:** 2020-02-13

**Authors:** Christopher L Ranque, Carol Stroble, Matthew J Amicucci, Diane Tu, Aly Diana, Sofa Rahmannia, Aghnia Husnayiani Suryanto, Rosalind S Gibson, Ying Sheng, Jennyfer Tena, Lisa A Houghton, Carlito B Lebrilla

**Affiliations:** 1 Department of Chemistry, University of California, Davis, CA, USA; 2 Department of Human Nutrition, University of Otago, Dunedin, New Zealand; 3 Nutrition Working Group, Faculty of Medicine, Universitas Padjadjaran, Bandung, Indonesia; 4 Faculty of Medicine, Universitas Pasundan, Bandung, Indonesia

**Keywords:** breast milk, feces, monosaccharide, oligosaccharide, LC-MS

## Abstract

**Background:**

The stable isotope deuterium dose-to-mother (DTM) technique to estimate nonbreast milk water intake demonstrates that maternal self-report methods of infant feeding overestimate the true prevalence of exclusively breastfeeding practices.

**Objective:**

We aimed to determine potential monosaccharide and oligosaccharide markers that distinguish between exclusively breastfed (EBF) versus nonexclusively breastfed (non-EBF) infants utilizing LC-MS-based methods.

**Methods:**

Data for the analysis were collected as part of a larger, longitudinal study of 192 breastfed Indonesian infants aged 2 mo and followed up at 5 mo. Feces samples were collected from infants aged 2 mo (*n* = 188) and 5 mo (*n* = 184). EBF and non-EBF strata at each time point were determined via the DTM technique. Feces samples were analyzed to determine monosaccharide content using ultra-high-performance LC-triple quadrupole MS (UHPLC-QqQ MS). Relative abundances of fecal oligosaccharides were determined using nano-LC-Chip-quadrupole time-of-flight MS (nano-LC-Chip-Q-ToF MS).

**Results:**

At age 2 mo, monosaccharide analysis showed the abundance of fructose and mannose were significantly higher (+377% and +388%, respectively) in non-EBF compared with EBF infants (*P* <0.0001). Fructose and mannose also showed good discrimination with areas under the curve (AUC) of 0.86 and 0.82, respectively. Oligosaccharide analysis showed that a 6-hexose (Hex_6_) isomer had good discrimination (AUC = 0.80) between EBF and non-EBF groups at 5 mo.

**Conclusion:**

Carbohydrate products, particularly fecal mono- and oligosaccharides, differed between EBF and non-EBF infants aged under 6 mo and can be used as potential biomarkers to distinguish EBF versus non-EBF feeding practices.

## Introduction

Breastfeeding has been shown to significantly reduce the incidence of infant disease and mortality (1–10). Specifically, prolonged breastfeeding for ≥6 mo has been shown to protect against diarrhea morbidity (11) and reduce the risk of infants contracting respiratory diseases (8). With its many benefits, the WHO recommends exclusive breastfeeding for ≥6 mo, defined as the infant receiving only breast milk, with no additional solids or liquids and water, along with continued breastfeeding for 2 y and beyond (12).

Efforts to increase exclusive breastfeeding rates globally are hampered by the ability to accurately assess breastfeeding practices. Maternal self-report is the easiest and most common method to estimate breastfeeding status (exclusive versus nonexclusive breastfed or EBF versus non-EBF) in the field on a global scale, yet accuracy is limited (13). Deuterated water (D_2_O) has been successfully used in lactating mothers to measure infant breast milk intake and exclusivity of breastfeeding practice (14–17). The D_2_O method (dose-to-mother; DTM) has been shown to be precise and accurate, and recent efforts to shorten the protocol have enhanced its usability in larger-scale surveys (17, 18). However, it is not without mother-infant burden, particularly given the collection of saliva samples over a 14-d period. As such, there is a need for other potential markers of exclusive breastfeeding practice, such as metabolites and proteins commonly found in complementary foods. To date, methods to determine these markers of infant feeding practices are limited.

Most complementary first foods consumed by infants are high in carbohydrates. Given that plant-based oligosaccharide structures are unique from human milk oligosaccharides (HMOs) found in breast milk, we have therefore examined carbohydrate products of infant digestion. Human milk is rich in carbohydrates, primarily lactose (composed of glucose and galactose), but also HMOs composed of glucose, galactose, *N*-acetylglucosamine (GlcNAc), fucose, and sialic acid (19–21). Monosaccharides represent a unique class of metabolites, well suited for biomarker discovery due to inherent differences between animal- and plant-based monosaccharides. Plant carbohydrates are composed primarily of glucose, fructose, mannose, and galacturonic acid (GalA) derived from larger polysaccharides such as starch, cellulose, pectins, and mannans (22, 23). Specific monosaccharides including fructose and GalA are generally found in plants but are not endogenous animal monosaccharides and may therefore differ between EBF and non-EBF infants. Indeed, it has earlier been suggested that the ratio of GlcNAc to endogenous mannose measured in urine may be a promising biomarker to determine breast milk intake (24).

The present study examines monosaccharides and plant based-oligosaccharides as potential biomarkers in infant feces in order to distinguish between EBF and non-EBF status designated using a state-of-the-art DTM method (17, 18). HMOs from breast milk are relatively abundant in the feces of breastfed infants and could interfere with the LC-MS analysis, but they have unique compositions and structures that have been characterized and annotated previously in our laboratory (21, 25). The unique monosaccharide compositions of HMOs include components that are neither found nor highly abundant in plants such as sialic acids and fucose. However, HMOs are not suitable candidates as biomarkers because all infants are continually breastfed, and the amounts of HMOs in feces depend on the amount of milk consumed and the state of the gut microbiota.

## Methods

Data for the analysis have been collected as part of a larger, longitudinal study of 192 breastfed Indonesian infants enrolled at age 2 mo and followed up at 5 mo. The overall aim of the study was to validate the shortened DTM protocol in a sample of EBF and non-EBF infants. D_2_O dosing and tissue sampling methods have been described previously (16–18). Based on the nonmilk water intake, infants were categorized into EBF or non-EBF status.

Feces samples from infants aged 2 mo (first visit) and 5 mo (second visit), were collected from Indonesian infants as described in the next section. During the 2-mo visit, 188 samples were collected from infants and classified as EBF or non-EBF based on DTM methods outlined by Liu et al. (17). In the 5-mo visit, 184 samples were collected and again given EBF or non-EBF designations from DTM results. The sample set was then defined for the 2-mo visit as *n* (EBF) = 160 and *n* (non-EBF) = 28, and for the 5-mo visit as *n* (EBF) = 86 and *n* (non-EBF) = 98. Monosaccharide standards and reagents used have been previously described by Xu et al. (26).

### Feces sample collection

Collection of feces occurred on 1 of the saliva sampling days within the DTM protocol previously described by Liu et al. (17). Approximately 5–10 mg of feces were collected into Eppendorf tubes and stored in ice-pack-cooled insulated boxes, then transferred to long-term storage in −80°C freezers.

### Ethics

Ethical approval was obtained from the Health Research Ethics Committee, Faculty of Medicine, Universitas Padjadjaran, Bandung, Indonesia (05/UN6.C1.3.2/KEPK/PN/2017). Parents or guardians gave informed written consent for the study.

### Monosaccharide and oligosaccharide extraction and clean-up from feces

For extraction, a slurry was made with a concentration of 100 mg dry feces per mL purified water. The extraction steps for feces have been previously described by De Leoz et al. (27). Samples were processed postextraction for clean-up with solid-phase extraction to further remove impurities, first with C8 followed by porous graphitized carbon. A more thorough description of the clean-up method has been reported by Ninonuevo et al. (28).

### Monosaccharide analysis sample preparation

This LC-MS-based method quantitates monosaccharides in feces as a measure of bioactivity attributed to the developing gut microbiota in infants. Rapid-throughput monosaccharide analysis was adapted from methods previously developed in our lab on 96-well plates from the methods of Xu et al. and Amicucci et al. (26, 29).

### Monosaccharide analysis by ultra-high-performance LC-triple quadrupole MS

The monosaccharides analyzed in this method are those that are unconjugated and not constituents of di-, oligo-, or polysaccharides. The separation of monosaccharides used a binary solvent system, solvent A contained 5% acetonitrile (ACN) and 25 mM ammonium acetate (NH_4_Ac) pH adjusted with ammonium hydroxide (NH_4_OH) to 8.2. Solvent B consisted of 95% ACN. A flow rate of 0.500 mL/min was used with the following gradient: 0–7 min, 12–15% B; 7–7.10 min, 15–99% B; 7.10–8.50 min, 99% B; 8.50–8.60 min, 99–12% B. Quantitation of monosaccharides in infant feces was conducted using a calibration curve constructed with a range from 1 µg to 100 mg monosaccharide per mg solution. The column used for separation was an Agilent ZORBAX RRHD ECLIPSE PLUS C18 column (150 × 2.1 mm) with 1.8 µm particle size. The UHPLC-QqQ system was an Agilent 1290 Infinity II UHPLC coupled to an Agilent 6495B QqQ mass spectrometer and 1 µL of each sample was injected for a run time of 10 min. Further details on instrument parameters and monosaccharide standards have been described by Xu et al. (26).

### Oligosaccharide analysis with nano-LC-Chip-quadrupole time-of-flight MS

Feces were analyzed for the presence of plant oligosaccharides, which are indicative of complementary feeding. Oligosaccharide analysis is far more complicated than monosaccharide analysis, thus, a subset of the original feces samples from EBF (*n* = 27) and non-EBF (*n* = 24) infants were examined for plant-based oligosaccharides at 2 mo and 5 mo. A binary solvent system was used where solvent A consisted of 3% ACN in 0.1% formic acid. Solvent B contained 90% ACN in 0.1% formic acid. The method used a 0.40 µL/min flow rate with the following gradient: 0–2.50 min, 1% B; 2.50–20.0 min, 1–16% B; 20.0–30.0 min, 16–44% B; 30.0–35.0 min, 44–99% B; 35.0–45.0 min, 99% B; 45.0–46.0 min, 99–0% B. Instrument parameter details have been previously described by Davis et al. (29). The structures of the oligosaccharides were not fully elucidated, however, they can be identified and characterized based on retention times and monosaccharide composition. Tandem MS was also used to confirm the oligosaccharide composition. Samples were quantified based on the comparison of peak ion counts. Nomenclature for oligosaccharide species was developed to distinguish between structures and isomers. For example, Hex_2_Pnt(b) would be an oligosaccharide comprised of 2 hexoses (6-carbon sugars) and 1 pentose (5-carbon sugar). The “(b)” designation means this oligosaccharide is the second eluting isomer of its species. As an additional example, Hex_11_ is an oligosaccharide comprised of 11 hexoses and the lack of a letter designation means this oligosaccharide was the only isomer detected.

### Statistical and data analysis

Statistical analyses were performed using GraphPad Prism 8 software (GraphPad Software, LLC). Monosaccharide and oligosaccharide analysis results were used with unpaired, 2-tailed t tests (significance defined as *P* <0.05; 2-sided) to determine the significance of differences observed between EBF versus non-EBF infants for mono- and oligosaccharide content. The t tests were conducted on each individual monosaccharide and oligosaccharide measurement and their totals at 2 mo. The t tests were also conducted at 5 mo on each individual monosaccharide and oligosaccharide measurement and their totals. Unpaired, 2-tailed t tests were also performed on oligosaccharide measurements within each respective group (EBF or non-EBF) to determine the significance of differences observed between the 2-mo and 5-mo visits. The unpaired t test was used to compare differences between 2 mo and 5 mo as a significant portion of participants’ breastfeeding status had changed between these time periods. Receiver operating characteristic (ROC) curves were constructed for each individual monosaccharide and oligosaccharide and their totals using EBF and non-EBF designations as the 2 distinguishing classes. AUCs were used to determine the discrimination capacity of each individual mono- and oligosaccharide, and total mono- and oligosaccharide content at ages 2 mo and 5 mo. Corresponding CIs (95% level), SEs, and *P* values for AUCs are also reported. The *P* value for an AUC tests the null hypothesis that the AUC is equal to 0.5. An AUC of 0.5 indicates a discrimination capacity equivalent to chance. Conversely, an AUC of 1.0 indicates a perfect test, with 100% probability of correctly distinguishing between classes.

## Results

### Analysis of fecal monosaccharides from EBF and non-EBF infants

**FIGURE 1:**
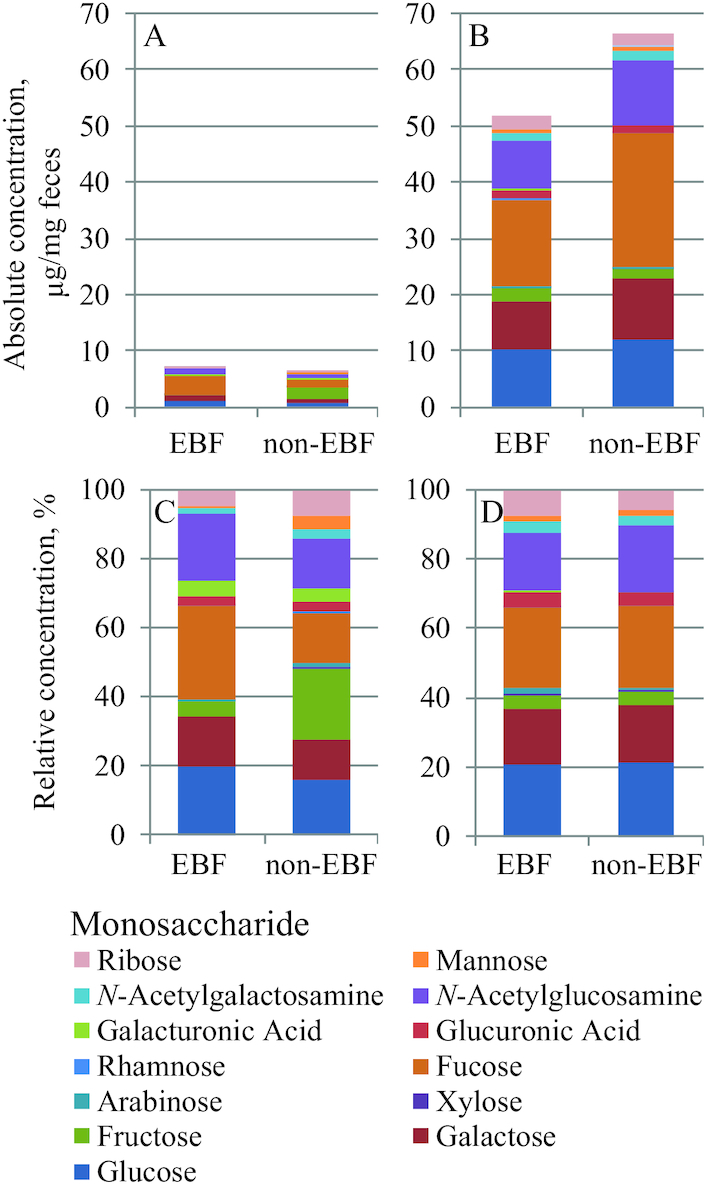
Comparison of endogenous monosaccharides in EBF versus non-EBF infants aged 2 mo (A, C) and 5 mo (B, D). Comparison of endogenous monosaccharides in EBF versus non-EBF infants aged 2 mo (A, C) and 5 mo (B, D). Absolute concentrations (abundances) are shown at 2 mo (A) and 5 mo (B), where relative concentrations are shown in the second row at 2 mo (C) and 5 mo (D). Values of stacked bars are mean concentrations of the monosaccharide measured, *n* (2 mo) = 160 (EBF) or 28 (non-EBF) and *n* (5 mo) = 86 (EBF) or 98 (non-EBF). Strata for infants were determined from the parent study based on the dose-to-mother technique as refined by Liu et al. (17). Key is read first from left to right and then top to bottom. This order corresponds to the appearance of the stacked bars in the graphs from top to bottom, e.g., glucose represents the bottom-most bar, galactose represents the second up from the bottom, fructose is the third bar up from the bottom, and ribose is the top-most bar. EBF and non-EBF infants consumed 812 mL/d and 329 mL/d of breastmilk during the 2-mo visit on average, and 942 mL/d and 564 mL/d during the 5-mo visit, respectively. EBF, exclusively breastfed; non-EBF, nonexclusively breastfed.

A summary of the fecal monosaccharides detected from both EBF and non-EBF infants is shown in **Figure 1**. At 2 mo (Figure 1A), both EBF and non-EBF infants had lower absolute abundances of fecal monosaccharides compared with 5 mo (Figure 1B). Analysis of the relative abundances showed marked differences between feces of EBF and non-EBF infants. EBF infants at 2 mo (Figure 1C) had considerably higher relative abundances of fecal fucose than non-EBF infants. Conversely, fecal samples for non-EBF infants were much higher in relative abundances of fructose compared with EBF infants. The results were consistent with the feeding behavior of both groups, as EBF infants were consuming only breast milk, which is rich in glucose, galactose, and fucose—the major monosaccharide components of human milk. Fructose is a major component of fruits and vegetables and would be observed in feces of non-EBF infants.

At 2 mo, the absolute abundances of fructose (*P* = 3.2 × 10^−9^), mannose (*P* = 6.4 × 10^−9^), and ribose (*P* = 0.032) were higher in the feces of non-EBF infants compared with EBF infants (**Supplemental Table 2**). For consistency, the relative abundances were compared, and those of fructose (*P* <0.0001), mannose (*P* <0.0001), arabinose (*P* = 0.0008), rhamnose (*P* = 0.012), and *N*-acetylgalactosamine (GalNAc) (*P* = 0.005) in feces were found to be higher for non-EBF infants than EBF infants at 2 mo. For EBF infants at 2 mo, absolute abundances of glucose (*P* = 0.048) were higher than non-EBF infants. Relative abundances of fucose (*P* = 0.003), galactose (*P* = 0.045), glucose (*P* = 0.029), and GlcNAc (*P* = 0.014) were also higher in feces of EBF infants than non-EBF infants at 2 mo (**Table 1**).

**TABLE 1:** Relative abundances of fecal monosaccharides for exclusively breastfed (*n* = 160) and nonexclusively breastfed (*n* = 28) infants aged 2 mo^1^

Monosaccharide	EBF	Non-EBF
Relative, %		
Fructose	4.4 ± 6.3	21 ± 20
Mannose	0.82 ± 1.2	4.0 ± 4.7
Fucose	27 ± 22	15 ± 14
Glucose	20 ± 906	16 ± 9.5
Galactose	14 ± 6.4	12 ± 6.6
*N*-Acetylgalactosamine	1.6 ± 1.7	2.8 ± 3.9
*N*-Acetylglucosamine	19 ± 10	14 ± 9.3
Ribose	4.3 ± 6.3	6.9 ± 9.9
Arabinose	0.56 ± 0.46	1.0 ± 1.5
Galacturonic acid	4.5 ± 4.0	3.9 ± 4.7
Rhamnose	0.10 ± 0.14	0.19 ± 0.31
Xylose	0.43 ± 0.34	0.45 ± 0.39
Glucuronic acid	3.0 ± 2.4	3.0 ± 2.8

1 Values are mean ± SD. EBF, exclusively breastfed; non-EBF, nonexclusively breastfed.

During the second visit at 5 mo, the absolute abundances of total fecal monosaccharides increased ≥5-fold compared with 2 mo (Figure 1A and B). This large increase of total fecal monosaccharides at 5 mo was observed in results for both EBF and non-EBF groups. Non-EBF infants had higher total absolute abundances of monosaccharides compared with EBF infants aged 5 mo (*P* = 0.043; **Supplemental Table 3**). For absolute abundances of individual monosaccharides at 5 mo, GlcNAc (*P* = 0.026) was higher in non-EBF infants compared with EBF infants. Xylose (*P* = 0.038) was higher in absolute abundances for EBF infants compared with non-EBF infants at 5 mo. Only the relative abundances of GalA (*P* = 0.049) were found to be higher in non-EBF infants at 5 mo (**Supplemental Table 1**); however, both xylose and GalA are monosaccharides mainly found in plants.

### Oligosaccharide analysis of feces

Between the ages of 2 mo to 5 mo, the infants’ gut microbiota matures and may process polysaccharides converting them to oligosaccharides. From the analysis, 31 plant-based oligosaccharides were found across all samples, as shown in **Supplemental Figure 2**, and quantified based on peak areas using ion counts. The oligosaccharides (and their isomers) detected were composed of hexose (Hex, C_6_H_12_O_6_) and pentose (Pnt, C_5_H_10_O_5_) monosaccharides.

At the age of 2 mo, the most abundant oligosaccharide was Hex_6_(b) for non-EBF infants, and Hex_5_(a) for EBF infants. For non-EBF infants at 2 mo, other markedly abundant oligosaccharides were Hex_3_(b), Hex_6_(c), Hex_4_(a), Hex_4_(b), Hex_4_(c), and Hex_4_(d). For EBF infants at 2 mo, other abundant oligosaccharides were Hex_6_(a), Hex_6_(b), Hex_4_(a), Hex_4_(b), Hex_4_(d), and Hex_11_. Pentose oligosaccharides were lowest in abundance compared with hexose-based oligosaccharides for both EBF and non-EBF infants at 2 mo. The most abundant pentose oligosaccharide present in EBF infant feces was Pnt_6_(a). For non-EBF infants, Pnt_4_ was the most abundant. Between EBF and non-EBF infants, it was found that abundances of Hex_4_(b) (*P* = 0.023) and Hex_4_(c) (*P* = 0.003) were significantly higher in non-EBF than in EBF infants at 2 mo (**Supplemental Table 4**). Total abundances of all plant-based oligosaccharides did not differ between EBF and non-EBF groups at the age of 2 mo (*P* = 0.97).

Observations at 5 mo differed compared with 2 mo. Notably, total plant-based oligosaccharide abundances in EBF infants were lower at 5 mo than 2 mo (*P* = 0.004; **Supplemental Table 6**). For non-EBF infants, total plant-based oligosaccharide abundances were higher at 5 mo than 2 mo, but these differences did not reach statistical significance (*P* = 0.39; **Supplemental Table 7**). Comparisons between the ages of 2 and 5 mo for EBF infants show that the Hex_3_Pnt (*P* = 0.029), Hex_4_Pnt(b) (*P* = 0.039), Hex_11_ (*P* = 0.036), and Hex_6_(a) (*P* = 0.033) oligosaccharides were significantly lower at 5 mo compared with 2 mo. For non-EBF infants, Pnt_4_ (*P* = 0.011), HexPnt_3_(b) (*P* = 0.034), Hex_4_Pnt(a) (*P* = 0.014), and Hex_5_(b) were significantly lower at 5 mo than at 2 mo (*P* = 0.011). However, abundances of Hex_5_(b) (*P* = 0.041) and Hex_7_(a) (*P* = 0.011) were significantly higher at 5 mo compared with 2 mo in non-EBF infants.

Observations at 5 mo alone showed that Hex_4_(b) was most abundant in non-EBF infants and Hex_4_(d) was most abundant in EBF infants. The Hex_3_(c) (*P* = 0.040), Hex_5_(b) (*P* = 0.013), and Hex_6_(a) (*P* = 0.031) oligosaccharides were found to be significantly higher in non-EBF infants than in EBF infants (**Supplemental Table 5**).

### Construction of ROC curves

We constructed ROC curves and examined AUCs to determine the potential of mono- and oligosaccharides as biomarkers to distinguish between EBF and non-EBF groups. Fructose and mannose had the highest AUCs of 0.86 and 0.82, respectively, for infants aged 2 mo (**Table 2**) and were found in higher relative abundances for non-EBF infants than EBF. Fucose was also found to have an AUC of 0.73 at 2 mo, which supports our earlier finding that abundances of fucose were significantly higher in EBF infants compared with non-EBF infants. AUCs of other monosaccharides, glucose, galactose, and GlcNAc were <0.70, a putative indication of low discrimination between non-EBF and EBF infants. Although these monosaccharides are major components in human milk, they are also found in plants and complementary foods. AUCs of the remaining monosaccharides GalNAc, ribose, arabinose, GalA, glucuronic acid (GlcA), rhamnose, and xylose also indicated low discrimination, with values <0.70. At 5 mo, nearly all monosaccharides tested had AUCs <0.70, which supported earlier observations that did not differentiate between EBF and non-EBF infants.

**TABLE 2:** Discrimination capacities of monosaccharide measurements using AUCs from receiver operating characteristic curves of exclusively breastfed (*n* = 160) and nonexclusively breastfed (*n* = 28) infants^1^

Monosaccharide	AUC	95% CI of the AUC	*P* value of the AUC
Fructose	0.86 ± 0.041	0.78–0.94	<0.0001
Mannose	0.82 ± 0.043	0.73–0.90	<0.0001
Fucose	0.73 ± 0.055	0.62–0.84	<0.0001
Glucose	0.67 ± 0.059	0.55–0.78	0.0046
Galactose	0.63 ± 0.059	0.51–0.74	0.034
*N*-Acetylgalactosamine	0.62 ± 0.055	0.51–0.73	0.045
*N*-Acetylglucosamine	0.62 ± 0.051	0.50–0.73	0.059
Ribose	0.60 ± 0.059	0.48–0.71	0.10
Arabinose	0.60 ± 0.065	0.47–0.72	0.11
Galacturonic acid	0.58 ± 0.066	0.45–0.71	0.19
Rhamnose	0.52 ± 0.066	0.39–0.65	0.75
Xylose	0.52 ± 0.063	0.39–0.64	0.77
Glucuronic acid	0.50 ± 0.063	0.38–0.63	0.93

1 Values are mean ± SEM. AUCs represent overall probabilities that indicate a measurement's capacity to discriminate between classes. An AUC above 0.80 indicates good discrimination between EBF and non-EBF groups. Conversely, an AUC of 0.50 signifies a measurement that is no better than chance at distinguishing between classes. Monosaccharides are listed from highest AUC to the lowest.

ROC curves were also constructed for plant-based oligosaccharides to determine their biomarker potential, using absolute abundances (Supplemental Tables 4 and 5). Hex_6_(a) also had the highest AUC of 0.80 at 5 mo. Interestingly, Hex_6_(a) at 2 mo yielded the highest AUC of 0.75. Furthermore, Hex_4_(c) at 2 mo yielded an AUC of 0.70, but at 5 mo had an AUC of 0.66. All other fecal oligosaccharides tested at both 2 mo and 5 mo did not distinguish between EBF and non-EBF groups (AUC <0.70).

## Discussion

Monosaccharide markers in feces provided good to excellent potential for biomarkers of complementary feeding. At 2 mo, higher fecal abundances of fructose and mannose for non-EBF infants differentiated this group from EBF infants. Both fructose and mannose had *P* values <0.05 when comparing EBF against non-EBF infants as well as high AUCs of 0.86 and 0.82. This is indicative of good discrimination between EBF and non-EBF infants and suggests complementary feeding of foods rich in fructose and mannose, likely from fruits. At age 2 mo, the infants’ microbiota may not have developed to efficiently utilize and break down plant sugars, thus these monosaccharides build-up and are passed in feces. Other monosaccharides, such as arabinose, rhamnose, and GalNAc are not found in HMOs, and were found in higher abundances in non-EBF infant feces than in EBF infant feces. However, they did not reach AUC levels >0.70, indicating low discrimination between EBF and non-EBF groups. Although galactose, GlcNAc, and glucose are common constituents in human milk, they had low discrimination between groups at 2 mo. This can be expected as these monosaccharides both exist in plants and HMOs and would be found in considerable amounts in both EBF and non-EBF groups. Fucose, an abundant monosaccharide in human milk, was more abundant in EBF infants at 2 mo, but had lower discrimination between EBF and non-EBF infants than fructose and mannose (AUC = 0.73). However, both EBF and non-EBF infants were breastfed so it is also expected that both groups had considerable amounts of fecal fucose. GlcA, GalA, and xylose were of similar abundances in both EBF and non-EBF infants, as indicated by a lack of statistical significance and low AUCs. These monosaccharides are also found in plants, such as in pectins as part of fruit cell walls. Ribose was also found in infant feces, likely as a product of metabolic processes (30). We suspect that differences between the 2 groups reflect differences in diet, the infants’ gastrointestinal development, and their gut microbial communities; however, these factors are not mutually exclusive.

At 5 mo, however, non-EBF infants’ gastrointestinal tracts have developed considerably to also utilize and break down plant monosaccharides (31). The monosaccharide analysis of fructose and mannose no longer distinguishes between EBF and non-EBF infants (AUCs <0.60; Supplemental Table 1). Furthermore, fucose discriminated between groups at 2 mo, but not at 5 mo (AUC = 0.51). The infants’ microbial communities are quickly developing during this period and would have a greater ability to process plant carbohydrates. Once their ability to convert plant-based polysaccharides into glucose or lactate becomes more efficient (32), fewer larger polymers are expected in feces. For example, at age 5 mo the infants’ increased consumption of glucose and galactose as nutrients, and increased usage of GlcNAc into glycans (e.g., glycosaminoglycans or proteoglycans [33]) could explain the deviation from observations at 2 mo. Fucose is unique to human milk and is released when HMOs are degraded (34, 35). Fucosylated HMOs have been shown to promote the growth of bifidobacteria (36–38), where we expect a more developed community of these commensals can be attributed to the decrease of fucose in feces.

Therefore, it is recommended that fructose and mannose serve as biomarkers at the age of 2 mo. The interplay between infant and gut microbial growth and increased consumption of breast milk and complementary foods presents a challenge in identifying a single group of biomarkers for EBF versus non-EBF indicators. We posit that change is attributable mainly to the increased utilization of carbohydrates by the infants and their microbiota. Previous studies have shown that an infant's gut microbiota develops to become functional within the first year (39–42), thus, it is reasonable the infants’ gut microbiota have adapted within our test window to process complementary foods.

However, both EBF and non-EBF groups at the ages of 2 mo and 5 mo reveal plant-based oligosaccharides present in their feces. Hexose- and pentose-based oligosaccharides were the most abundant found in the infants’ feces. Hexose-based oligosaccharides are potentially derived from various plant-based sources, the most common being starch and cellulose (43). One of the most notable hexose oligosaccharides was Hex_6_(a), which was significantly higher in EBF infants at 2 mo with an AUC of 0.75, indicating fair discrimination between EBF and non-EBF infants. However, at 5 mo Hex_6_(a) was significantly higher in non-EBF infants with greater discrimination (AUC = 0.80). Hex_6_(a) is interesting as it is probably derived from starchy fruits and vegetables and is likely to be an α(1,4)-glucose oligomers, potentially from complementary foods. Other common plant polysaccharides such as xyloglucan are also potential sources of hexose-based oligosaccharides with pentose decorations (44), e.g., Hex_3_Pnt. Larger pentose-based oligosaccharides are likely derived from arabinoxylan or heteroxylans, such as HexPnt_3_(a), HexPnt_3_(b), and HexPnt_3_(c), with hexose decorations (45).

Although the presence of plant-based oligosaccharides likely originates from complementary food, there were unexpected amounts found in EBF infant feces. There are some likely explanations, perhaps the EBF infants were not rigorously EBF or these oligosaccharides came from shedding by the gut microbes; however, neither could be confirmed in the study. Because the cut-off for nonmilk water intake that stratified infants into EBF and non-EBF groups is not zero, there is a reasonable amount of inherent dietary flexibility for participants. However, the presence of plant-based oligosaccharides in EBF infant feces does not necessarily mean the infant did not receive the benefits of the breastfeeding regimen. The review by Kramer et al. on the optimal duration of breastfeeding found that those studies did not strictly adhere to the WHO definition, despite evidence supporting the recommendation for 6 mo exclusive breastfeeding (46).

We expect the variations observed in our data are attributed to diet. However, our expectation that common complementary foods would be high in starch and cellulose is corroborated by the abundance of hexose-based oligosaccharides. Furthermore, for the purposes of developing methods towards assessing breastfeeding practices globally it is more pragmatic to evaluate the infants’ feeding patterns as would be done in the absence of supervision. To evaluate the consumption of plant-based sugars collectively, total absolute abundances of all observed oligosaccharides were calculated for each infant and averaged within their respective groups (EBF or non-EBF). Total abundances of plant-based oligosaccharides were compared between EBF and non-EBF infants at 2 mo and compared again at 5 mo as a potential biomarker. However, the total abundances of plant-based oligosaccharides at 2 mo (*P* = 0.972) and 5 mo (*P* = 0.053) were not statistically significant (Supplemental Tables 4 and 5). At 2 mo, mean total plant-based oligosaccharide abundances were comparable between EBF and non-EBF groups. However, it is currently unknown whether non-EBF infants would develop the ability to digest complementary foods faster than EBF infants in view of their greater consumption during the first few months after birth. For example, Hex_6_(b) was highly abundant in non-EBF infants at 2 mo, but at 5 mo Hex6(b) abundances dropped significantly (*P* = 0.04; Supplemental Table 7). This observation is further supported by the increase of smaller hexose-based oligosaccharides such as Hex_4_(a), Hex_4_(b), Hex_4_(c), and Hex_4_(d), derived from the break down of larger poly- and oligosaccharides. A decrease in larger oligosaccharides coupled with increased amounts of smaller oligosaccharides indicates more efficient digestion. Based on the demographic and dietary patterns represented in this study, it is recommended to use Hex_6_(a) as a biomarker at the age of 5 mo to distinguish between EBF and non-EBF infants. Additional work would be necessary to explore the impact of diet and state of health of these infants on the variation observed within these samples. Although the technology and methods utilized in this study are far more sophisticated than maternal self-report, they address the issue of subjectivity and present a lower mother-infant burden on sample collection inherent to DTM. Feces samples can be collected as needed, and do not require prior administration of isotope-labeled materials. This method is not intended to supplant DTM, rather it serves as a complementary method in its validation. However, the greater challenge is in determining markers that can persist in even larger sample sets representing a broad population to more accurately measure breastfeeding practices globally.

## Supplementary Material

nxaa028_Supplemental_File

## Notes

Supported by the Bill and Melinda Gates Foundation (Grant No. OPP1127870), as part of the “Developing biomarkers of exclusive breastfeeding practice” project. The Bill and Melinda Gates Foundation did not have a role in the design, implementation, analysis, or interpretation of the data.

Author disclosures: The authors report no conflicts of interest.

Supplemental Tables 1–7 and Supplemental Figures 1–3 are available from the “Supplementary data” link in the online posting of the article and from the same link in the online table of contents at https://academic.oup.com/jn/.

Abbreviations used: D_2_O, deuterium oxide; DTM, dose-to-mother; EBF, exclusively breastfed; GalA, galacturonic acid; GalNAc, *N*-acetylgalactosamine; GlcA, glucuronic acid; GlcNAc, *N*-acetylglucosamine; Hex, hexose; HMO, human milk oligosaccharide; non-EBF, nonexclusively breastfed; Pnt, pentose; ROC, receiver operating characteristic.
