# Supplementary material for: Examination of Carbohydrate Products in Feces Reveals Potential Biomarkers Distinguishing Exclusive and Nonexclusive Breastfeeding Practices in Infants
Source: J Nutr. 2020 Feb 13;150(5):1051–7. doi: 10.1093/jn/nxaa028 (PMC7198307; doi:10.1093/jn/nxaa028)
Supplement: nxaa028_Supplemental_File [file nxaa028_supplemental_file.docx]

Online Supplementary Material for “Examination of carbohydrate products in feces reveals potential biomarkers distinguishing exclusive and non-exclusive breastfeeding practices in infants”

Authors: Christopher L. Ranque, Carol Stroble, Matthew J. Amicucci, Diane Tu, Aly Diana, Sofa Rahmannia, Aghnia Husnayiani Suryanto, Rosalind S. Gibson, Ying Sheng, Jennyfer Tena, Lisa A. Houghton, Carlito B. Lebrilla

**Supplemental Table 1. Relative Free Monosaccharide Analysis results for 5 months visit^1^.**

| Monosaccharide | EBF, % | Non-EBF, % | | Unpaired *t*-test *P*-value | | AUC^2^ | AUC 95% CI | | AUC *P*-value |
| --- | --- | --- | --- | --- | --- | --- | --- | --- | --- |
| Fructose | 3.8 ± 8.2 | | 4.1 ± 9.3 | 0.958 | 0.50 ± 0.043 | | | 0.42 – 0.59 | 0.973 |
| Mannose | 1.6 ± 2.6 | | 1.5 ± 2.9 | 0.112 | 0.52 ± 0.043 | | | 0.44 – 0.60 | 0.645 |
| Fucose | 23 ± 17 | | 24 ± 20 | 0.424 | 0.51 ± 0.043 | | | 0.42 – 0.59 | 0.889 |
| Glucose | 20 ± 13 | | 21 ± 16 | 0.167 | 0.51 ± 0.043 | | | 0.43 – 0.60 | 0.773 |
| Galactose | 16 ± 7.1 | | 16 ± 7.0 | 0.881 | 0.50 ± 0.043 | | | 0.42 – 0.59 | 0.947 |
| *N*-Acetylgalactosamine | 3.5 ± 3.1 | | 3.2 ± 2.7 | 0.136 | 0.52 ± 0.043 | | | 0.44 – 0.61 | 0.598 |
| *N*-Acetylglucosamine | 16 ± 10 | | 19 ± 11 | 0.851 | 0.57 ± 0.042 | | | 0.49 – 0.66 | 0.089 |
| Ribose | 7.5 ± 8.2 | | 5.6 ± 4.9 | 0.890 | 0.55 ± 0.043 | | | 0.47 – 0.63 | 0.235 |
| Arabinose | 1.3 ± 2.5 | | 0.88 ± 1.6 | 0.681 | 0.56 ± 0.042 | | | 0.47 – 0.64 | 0.192 |
| Galacturonic acid | 0.31 ± 0.70 | | 0.34 ± 2.0 | 0.049 | 0.53 ± 0.043 | | | 0.44 – 0.61 | 0.546 |
| Rhamnose | 0.22 ± 0.33 | | 0.14 ± 0.19 | 0.051 | 0.56 ± 0.043 | | | 0.48 – 0.65 | 0.133 |
| Xylose | 0.52 ± 0.64 | | 0.35 ± 0.44 | 0.903 | 0.62 ± 0.041 | | | 0.54 – 0.70 | 0.005 |
| Glucuronic acid | 4.5 ± 5.3 | | 3.5 ± 4.1 | 0.403 | 0.57 ± 0.043 | | | 0.48 – 0.65 | 0.127 |

^1^EBF, % and Non-EBF, %: Values are mean ± SD, *n* = 86 (EBF) or 98 (non-EBF) infants. AUC, area under the receiver operating characteristic curve; EBF, exclusive breastfed; Non-EBF, non-exclusively breastfed.

^2^AUC: Values are mean ± SEM, *n* = 86 (EBF) or 98 (non-EBF) infants.

**Supplemental Table 2. Absolute Free Monosaccharide Analysis results for 2 months visit^1^.**

| Monosaccharide | EBF, µg/mg feces | Non-EBF, µg/mg feces | Unpaired *t*-test *P*-value | AUC^2^ | AUC 95% CI | AUC *P*-value |
| --- | --- | --- | --- | --- | --- | --- |
| Fructose | 0.19 ± 0.29 | 2.04 ± 3.76 | 3.16E-09 | 0.70 ± 0.068 | 0.57 – 0.84 | 0.0006 |
| Mannose | 0.041 ± 0.075 | 0.36 ± 0.64 | 6.38E-09 | 0.69 ± 0.066 | 0.56 – 0.82 | 0.001 |
| Fucose | 3.24 ± 6.58 | 1.34 ± 2.71 | 0.116 | 0.69 ± 0.058 | 0.57 – 0.80 | 0.002 |
| Glucose | 1.17 ± 0.91 | 0.83 ± 0.63 | 0.048 | 0.63 ± 0.056 | 0.52 – 0.74 | 0.027 |
| Galactose | 0.84 ± 0.73 | 0.60 ± 0.47 | 0.076 | 0.61 ± 0.058 | 0.49 – 0.72 | 0.070 |
| *N*-Acetylgalactosamine | 0.081 ± 0.089 | 0.088 ± 0.074 | 0.684 | 0.58 ± 0.053 | 0.47 – 0.68 | 0.201 |
| *N*-Acetylglucosamine | 1.10 ± 0.96 | 0.75 ± 0.62 | 0.052 | 0.61 ± 0.061 | 0.49 – 0.73 | 0.071 |
| Ribose | 0.15 ± 0.097 | 0.20 ± 0.14 | 0.032 | 0.62 ± 0.056 | 0.51 – 0.73 | 0.041 |
| Arabinose | 0.025 ± 0.013 | 0.030 ± 0.018 | 0.088 | 0.61 ± 0.058 | 0.49 – 0.72 | 0.068 |
| Galacturonic acid | 0.22 ± 0.20 | 0.19 ± 0.24 | 0.535 | 0.55 ± 0.066 | 0.42 – 0.68 | 0.409 |
| Rhamnose | 0.0042 ± 0.0028 | 0.0046 ± 0.0041 | 0.454 | 0.53 ± 0.060 | 0.41 – 0.65 | 0.627 |
| Xylose | 0.018 ± 0.011 | 0.014 ± 0.0073 | 0.077 | 0.57 ± 0.055 | 0.46 – 0.67 | 0.264 |
| Glucuronic acid | 0.14 ± 0.12 | 0.13 ± 0.14 | 0.788 | 0.52 ± 0.066 | 0.39 – 0.65 | 0.718 |
| Total | 7.23 ± 7.53 | 6.60 ± 6.43 | 0.662 | --- | --- | --- |

^1^EBF, µg/mg feces and Non-EBF, µg/mg feces: Values are mean ± SD, *n* = 160 (EBF) or 28 (non-EBF) infants. AUC, area under the receiver operating characteristic curve; EBF, exclusive breastfed; Non-EBF, non-exclusively breastfed.

^2^AUC: Values are mean ± SEM, *n* = 160 (EBF) or 28 (non-EBF) infants.

**Supplemental Table 3. Absolute Free Monosaccharide Analysis results for 5 months visit^1^.**

| Monosaccharide | EBF, µg/mg feces | Non-EBF, µg/mg feces | Unpaired *t*-test *P*-value | AUC^2^ | AUC 95% CI | AUC *P*-value |
| --- | --- | --- | --- | --- | --- | --- |
| Fructose | 2.11 ± 6.34 | 1.87 ± 4.79 | 0.769 | 0.57 ± 0.043 | 0.48 – 0.65 | 0.126 |
| Mannose | 0.74 ± 1.67 | 0.80 ± 1.96 | 0.817 | 0.52 ± 0.043 | 0.44 – 0.61 | 0.594 |
| Fucose | 15.27 ± 21.09 | 23.55 ± 39.71 | 0.085 | 0.53 ± 0.043 | 0.45 – 0.61 | 0.466 |
| Glucose | 10.49 ± 9.39 | 12.16 ± 13.42 | 0.338 | 0.55 ± 0.043 | 0.47 – 0.63 | 0.243 |
| Galactose | 8.46 ± 7.48 | 10.62 ± 9.61 | 0.095 | 0.57 ± 0.042 | 0.49 – 0.65 | 0.110 |
| *N*-Acetylgalactosamine | 1.41 ± 1.35 | 1.50 ± 1.21 | 0.644 | 0.54 ± 0.043 | 0.45 – 0.62 | 0.376 |
| *N*-Acetylglucosamine | 8.56 ± 8.50 | 11.65 ± 9.94 | 0.026 | 0.60 ± 0.042 | 0.52 – 0.68 | 0.017 |
| Ribose | 2.29 ± 1.47 | 2.13 ± 1.12 | 0.423 | 0.51 ± 0.043 | 0.43 – 0.59 | 0.829 |
| Arabinose | 0.42 ± 0.81 | 0.31 ± 0.57 | 0.268 | 0.51 ± 0.043 | 0.42 – 0.59 | 0.863 |
| Galacturonic acid | 0.14 ± 0.56 | 0.089 ± 0.34 | 0.449 | 0.50 ± 0.043 | 0.42 – 0.58 | >0.999 |
| Rhamnose | 0.069 ± 0.083 | 0.052 ± 0.043 | 0.073 | 0.51 ± 0.043 | 0.43 – 0.59 | 0.829 |
| Xylose | 0.22 ± 0.41 | 0.13 ± 0.11 | 0.038 | 0.55 ± 0.043 | 0.46 – 0.63 | 0.288 |
| Glucuronic acid | 1.61 ± 1.54 | 1.40 ± 1.08 | 0.278 | 0.50 ± 0.043 | 0.42 – 0.59 | 0.916 |
| Total | 51.87 ± 40.48 | 66.43 ± 54.31 | 0.043 | --- | --- | --- |

^1^EBF, µg/mg feces and Non-EBF, µg/mg feces: Values are mean ± SD, *n* = 86 (EBF) or 98 (non-EBF) infants. AUC, area under the receiver operating characteristic curve; EBF, exclusive breastfed; Non-EBF, non-exclusively breastfed.

^2^AUC: Values are mean ± SEM, *n* = 86 (EBF) or 98 (non-EBF) infants.

**Supplemental Table 4. Comparison of plant-based oligosaccharides in EBF versus non-EBF infants 2 months^1^.**

| Oligosaccharide | EBF Peak Area, Ion Count | Non-EBF Peak Area, Ion Count | Unpaired *t*-test *P*-value | AUC^2^ | AUC 95% CI | AUC *P*-value |
| --- | --- | --- | --- | --- | --- | --- |
| Hex_3_(a) | 26696.15 ± 70506.13 | 25364.23 ± 78975.04 | 0.950 | 0.54 ± 0.082 | 0.38 – 0.70 | 0.597 |
| Hex_3_(b) | 51940.85 ± 128495.05 | 99514.77 ± 297937.25 | 0.454 | 0.58 ± 0.083 | 0.41 – 0.74 | 0.350 |
| Hex_3_(c) | 14940.35 ± 38462.37 | 33302.86 ± 84729.49 | 0.315 | 0.60 ± 0.085 | 0.43 – 0.76 | 0.235 |
| Pnt_4_ | 38413.52 ± 46195.36 | 44499.05 ± 39692.05 | 0.618 | 0.54 ± 0.087 | 0.36 – 0.71 | 0.664 |
| HexPnt_3_(a) | 9402.73 ± 11912.063 | 25038.52 ± 50163.61 | 0.122 | 0.53 ± 0.083 | 0.37 – 0.69 | 0.699 |
| HexPnt_3_(b) | 22839.92 ± 54466.83 | 10144.00 ± 18264.07 | 0.282 | 0.55 ± 0.082 | 0.39 – 0.71 | 0.552 |
| HexPnt_3_(c) | 48939.74 ± 71261.94 | 33381.24 ± 31207.04 | 0.328 | 0.55 ± 0.082 | 0.39 – 0.71 | 0.527 |
| Hex_3_Pnt | 16907.04 ± 23973.59 | 13784.29 ± 47920.20 | 0.766 | 0.69 ± 0.077 | 0.53 – 0.83 | 0.029 |
| Hex_4_(a) | 164762.89 ± 364525.02 | 93559.58 ± 159151.27 | 0.381 | 0.53 ± 0.084 | 0.37 – 0.70 | 0.678 |
| Hex_4_(b) | 64726.15 ± 73633.33 | 165324.83 ± 208991.21 | 0.023 | 0.61 ± 0.082 | 0.45 – 0.77 | 0.163 |
| Hex_4_(c) | 19696.19 ± 27821.40 | 59241.67 ± 58153.16 | 0.003 | 0.70 ± 0.081 | 0.54 – 0.86 | 0.014 |
| Hex_4_(d) | 89970.52 ± 117890.32 | 141050.67 ± 224535.89 | 0.307 | 0.52 ± 0.084 | 0.35 – 0.68 | 0.850 |
| Pnt_5_(a) | 4293.93 ± 4442.09 | 11294.33 ± 23492.49 | 0.135 | 0.54 ± 0.084 | 0.38 – 0.71 | 0.591 |
| Pnt_5_(b) | 5892.92 ± 14131.24 | 3841.43 ± 4252.41 | 0.498 | 0.50 ± 0.082 | 0.34 – 0.67 | 0.955 |
| Pnt_5_(c) | 3381.84 ± 3447.47 | 12671.43 ± 40762.97 | 0.243 | 0.54 ± 0.082 | 0.38 – 0.70 | 0.637 |
| Hex_4_Pnt(a) | 28216.07 ± 82121.56 | 26006.67 ± 40418.46 | 0.905 | 0.55 ± 0.083 | 0.38 – 0.71 | 0.578 |
| Hex_4_Pnt(b) | 13007.62 ± 13985.46 | 21269.15 ± 47836.75 | 0.395 | 0.58 ± 0.083 | 0.42 – 0.75 | 0.299 |
| Pnt_6_(a) | 55056.77 ± 175934.24 | 10064.50 ± 10464.04 | 0.217 | 0.62 ± 0.079 | 0.46 – 0.77 | 0.146 |
| Pnt_6_(b) | 6121.84 ± 7786.26 | 8251.76 ± 11615.84 | 0.441 | 0.50 ± 0.083 | 0.34 – 0.67 | 0.970 |
| Hex_5_(a) | 293462.85 ± 695366.26 | 42963.55 ± 68184.42 | 0.085 | 0.67 ± 0.077 | 0.51 – 0.82 | 0.044 |
| Hex_5_(b) | 47701.74 ± 76279.77 | 29175.70 ± 22562.91 | 0.258 | 0.61 ± 0.080 | 0.46 – 0.77 | 0.168 |
| Hex_11_ | 111684.60 ± 257630.99 | 47894.53 ± 86206.78 | 0.253 | 0.57 ± 0.082 | 0.41 – 0.73 | 0.423 |
| Hex_12_(a) | 30589.60 ± 85054.03 | 22120.00 ± 59201.85 | 0.685 | 0.62 ± 0.079 | 0.46 – 0.77 | 0.146 |
| Hex_12_(b) | 7153.23 ± 18683.13 | 9259.50 ± 12112.50 | 0.640 | 0.57 ± 0.088 | 0.40 – 0.75 | 0.365 |
| Hex_6_(a) | 92274.93 ± 195728.70 | 18158.30 ± 18273.22 | 0.071 | 0.75 ± 0.068 | 0.62 – 0.89 | 0.002 |
| Hex_6_(b) | 144891.81 ± 415107.36 | 450037.65 ± 985453.30 | 0.148 | 0.52 ± 0.080 | 0.36 – 0.69 | 0.763 |
| Hex_6_(c) | 24872.05 ± 72765.02 | 76277.95 ± 147465.72 | 0.115 | 0.62 ± 0.082 | 0.46 – 0.78 | 0.157 |
| Hex_7_(a) | 15148.75 ± 19160.46 | 20805.11 ± 21117.79 | 0.321 | 0.59 ± 0.081 | 0.43 – 0.75 | 0.278 |
| Hex_7_(b) | 36273.00 ± 51680.28 | 27928.21 ± 23350.78 | 0.471 | 0.56 ± 0.082 | 0.40 – 0.72 | 0.450 |
| Hex_7_(c) | 26393.65 ± 58767.75 | 26400.95 ± 61879.01 | 0.999 | 0.56 ± 0.081 | 0.40 – 0.72 | 0.450 |
| Hex_7_(d) | 18414.00 ± 41139.36 | 36299.11 ± 76519.61 | 0.296 | 0.62 ± 0.080 | 0.46 – 0.77 | 0.152 |
| Total | 1459983.07 ± 833705.59 | 1450651.92 ± 1080822.24 | 0.972 | 0.55 ± 0.083 | 0.39 – 0.71 | 0.559 |

^1^EBF Peak Area, Ion Count and Non-EBF Peak Area, Ion Count: Values are mean ± SD, *n* = 27 (EBF) or 24 (non-EBF) infants. AUC, area under the receiver operating characteristic curve; EBF, exclusive breastfed; Hex, hexose; Non-EBF, non-exclusively breastfed; Pnt, pentose.

^2^AUC: Values are mean ± SEM, *n* = 27 (EBF) or 24 (non-EBF) infants.

**Supplemental Table 5. Comparison of plant-based oligosaccharides in EBF versus non-EBF infants 5 months^1^.**

| Oligosaccharide | EBF Peak Area, Ion Count | Non-EBF Peak Area, Ion Count | Unpaired *t*-test *P*-value | AUC^2^ | AUC 95% CI | AUC *P*-value |
| --- | --- | --- | --- | --- | --- | --- |
| Hex_3_(a) | 20537.07 ± 69358.98 | 119946.70 ± 416477.40 | 0.218 | 0.67 ± 0.074 | 0.52 – 0.81 | 0.032 |
| Hex_3_(b) | 44336.14 ± 175324.70 | 103766.50 ± 263017.30 | 0.327 | 0.60 ± 0.078 | 0.44 – 0.75 | 0.219 |
| Hex_3_(c) | 4792.82 ± 13796.01 | 56829.56 ± 129548.50 | 0.040 | 0.67 ± 0.075 | 0.53 – 0.82 | 0.026 |
| Pnt_4_ | 18637.39 ± 58411.77 | 12988.48 ± 14638.93 | 0.628 | 0.57 ± 0.078 | 0.42 – 0.72 | 0.368 |
| HexPnt_3_(a) | 7252.61 ± 8385.28 | 6771.78 ± 4756.93 | 0.796 | 0.56 ± 0.081 | 0.40 – 0.71 | 0.474 |
| HexPnt_3_(b) | 4412.00 ± 8147.28 | 2442.04 ± 2004.64 | 0.228 | 0.54 ± 0.080 | 0.38 – 0.70 | 0.614 |
| HexPnt_3_(c) | 27070.61 ± 41285.59 | 19677.78 ± 21599.33 | 0.412 | 0.51 ± 0.080 | 0.35 – 0.67 | 0.886 |
| Hex_3_Pnt | 959.50 ± 1667.87 | 1107.41 ± 1703.52 | 0.746 | 0.51 ± 0.080 | 0.35 – 0.67 | 0.893 |
| Hex_4_(a) | 78096.36 ± 146650.70 | 283882.40 ± 557849.00 | 0.065 | 0.63 ± 0.077 | 0.47 – 0.78 | 0.110 |
| Hex_4_(b) | 33979.46 ± 42346.11 | 389286.60 ± 1396330.00 | 0.184 | 0.67 ± 0.074 | 0.53 – 0.81 | 0.031 |
| Hex_4_(c) | 25545.82 ± 53617.45 | 79335.89 ± 137742.60 | 0.060 | 0.66 ± 0.075 | 0.51 – 0.81 | 0.043 |
| Hex_4_(d) | 180241.30 ± 241413.20 | 317267.60 ± 490291.20 | 0.192 | 0.58 ± 0.078 | 0.43 – 0.74 | 0.285 |
| Pnt_5_(a) | 5427.93 ± 7479.22 | 6432.15 ± 9810.96 | 0.671 | 0.53 ± 0.079 | 0.37 – 0.68 | 0.736 |
| Pnt_5_(b) | 6056.15 ± 12791.59 | 6059.04 ± 13226.67 | 0.999 | 0.55 ± 0.079 | 0.39 – 0.70 | 0.561 |
| Pnt_5_(c) | 11955.93 ± 38911.26 | 9327.70 ± 14318.33 | 0.743 | 0.64 ± 0.076 | 0.49 – 0.79 | 0.077 |
| Hex_4_Pnt(a) | 10890.36 ± 26933.95 | 5993.00 ± 5856.34 | 0.360 | 0.56 ± 0.079 | 0.41 – 0.72 | 0.419 |
| Hex_4_Pnt(b) | 3683.71 ± 3853.26 | 9367.41 ± 28101.00 | 0.294 | 0.55 ± 0.079 | 0.39 – 0.70 | 0.567 |
| Pnt_6_(a) | 6079.14 ± 6356.05 | 6491.62 ± 6659.42 | 0.815 | 0.50 ± 0.079 | 0.35 – 0.66 | 0.973 |
| Pnt_6_(b) | 7613.04 ± 23509.89 | 4075.24 ± 5464.40 | 0.450 | 0.54 ± 0.079 | 0.39 – 0.70 | 0.579 |
| Hex_5_(a) | 72724.50 ± 131607.90 | 241622.60 ± 892993.20 | 0.327 | 0.56 ± 0.078 | 0.40 – 0.71 | 0.469 |
| Hex_5_(b) | 21204.20 ± 31036.28 | 80720.48 ± 117979.70 | 0.013 | 0.69 ± 0.073 | 0.54 – 0.83 | 0.018 |
| Hex_11_ | 6590.77 ± 27372.47 | 1157.83 ± 836.45 | 0.307 | 0.64 ± 0.076 | 0.50 – 0.79 | 0.067 |
| Hex_12_(a) | 19465.94 ± 43341.53 | 5918.35 ± 10386.46 | 0.120 | 0.54 ± 0.079 | 0.38 – 0.69 | 0.625 |
| Hex_12_(b) | 2800.60 ± 3951.56 | 2784.77 ± 2816.40 | 0.987 | 0.57 ± 0.078 | 0.41 – 0.72 | 0.399 |
| Hex_6_(a) | 11054.28 ± 9184.32 | 121174.30 ± 262313.60 | 0.031 | 0.80 ± 0.061 | 0.68 – 0.92 | 0.0001 |
| Hex_6_(b) | 45685.28 ± 149944.60 | 45022.23 ± 138678.40 | 0.987 | 0.60 ± 0.078 | 0.44 – 0.75 | 0.225 |
| Hex_6_(c) | 63583.67 ± 286525.70 | 36103.52 ± 118169.50 | 0.646 | 0.62 ± 0.077 | 0.46 – 0.77 | 0.143 |
| Hex_7_(a) | 62670.00 ± 138811.20 | 73398.62 ± 94788.04 | 0.740 | 0.67 ± 0.074 | 0.52 – 0.82 | 0.031 |
| Hex_7_(b) | 57747.96 ± 131790.00 | 48832.32 ± 83422.30 | 0.767 | 0.53 ± 0.079 | 0.38 – 0.69 | 0.662 |
| Hex_7_(c) | 29514.79 ± 67667.63 | 42105.12 ± 123181.10 | 0.639 | 0.53 ± 0.079 | 0.38 – 0.69 | 0.680 |
| Hex_7_(d) | 10832.95 ± 13331.85 | 9613.78 ± 11044.92 | 0.714 | 0.54 ± 0.079 | 0.39 – 0.70 | 0.590 |
| Total | 821636.32 ± 751828.25 | 2046176.15 ± 3180505.45 | 0.053 | 0.63 ± 0.076 | 0.48 – 0.78 | 0.106 |

^1^EBF Peak Area, Ion Count and Non-EBF Peak Area, Ion Count: Values are mean ± SD, *n* = 28 (EBF) or 27 (non-EBF) infants. AUC, area under the receiver operating characteristic curve; EBF, exclusive breastfed; Hex, hexose; Non-EBF, non-exclusively breastfed; Pnt, pentose.

^2^AUC: Values are mean ± SEM, *n* = 28 (EBF) or 27 (non-EBF) infants.

**Supplemental Table 6. Comparison of 2 months vs. 5 months of the absolute abundances of plant-based oligosaccharides for EBF Infants^1^.**

| Oligosaccharide | Peak Area at 2 months, Ion Count | Peak Area at 5 months, Ion Count | Unpaired *t*-test *P*-value |
| --- | --- | --- | --- |
| Hex_3_(a) | 26696.15 ± 70506.13 | 20537.07 ± 69358.98 | 0.745 |
| Hex_3_(b) | 51940.85 ± 128495.05 | 44336.14 ± 175324.70 | 0.856 |
| Hex_3_(c) | 14940.35 ± 38462.37 | 4792.821 ± 13796.01 | 0.195 |
| Pnt_4_ | 38413.52 ± 46195.36 | 18637.39 ± 58411.77 | 0.171 |
| HexPnt_3_(a) | 9402.73 ± 11912.06 | 7252.61 ± 8385.28 | 0.441 |
| HexPnt_3_(b) | 22839.92 ± 54466.83 | 4412.00 ± 8147.28 | 0.082 |
| HexPnt_3_(c) | 48939.74 ± 71261.94 | 27070.61 ± 41285.59 | 0.168 |
| Hex_3_Pnt | 16907.04 ± 23973.59 | 959.50 ± 1667.87 | 0.029 |
| Hex_4_(a) | 164762.89 ± 364525.02 | 78096.36 ± 146650.70 | 0.249 |
| Hex_4_(b) | 64726.15 ± 73633.33 | 33979.46 ± 42346.11 | 0.062 |
| Hex_4_(c) | 19696.19 ± 27821.40 | 25545.82 ± 53617.45 | 0.616 |
| Hex_4_(d) | 89970.52 ± 117890.32 | 180241.30 ± 241413.20 | 0.086 |
| Pnt_5_(a) | 4293.93 ± 4442.09 | 5427.93 ± 7479.22 | 0.499 |
| Pnt_5_(b) | 5892.92 ± 14131.24 | 6056.15 ± 12791.59 | 0.964 |
| Pnt_5_(c) | 3381.84 ± 3447.47 | 11955.93 ± 38911.26 | 0.260 |
| Hex_4_Pnt(a) | 28216.07 ± 82121.56 | 10890.36 ± 26933.95 | 0.294 |
| Hex_4_Pnt(b) | 13007.62 ± 13985.46 | 3683.71 ± 3853.26 | 0.039 |
| Pnt_6_(a) | 55056.77 ± 175934.24 | 6079.14 ± 6356.05 | 0.147 |
| Pnt_6_(b) | 6121.84 ± 7786.26 | 7613.04 ± 23509.89 | 0.756 |
| Hex_5_(a) | 293462.85 ± 695366.26 | 72724.50 ± 131607.90 | 0.105 |
| Hex_5_(b) | 47701.74 ± 76279.77 | 21204.20 ± 31036.28 | 0.095 |
| Hex_11_ | 111684.60 ± 257630.99 | 6590.77 ± 27372.47 | 0.036 |
| Hex_12_(a) | 30589.60 ± 85054.03 | 19465.94 ± 43341.53 | 0.542 |
| Hex_12_(b) | 7153.23 ± 18683.13 | 2800.60 ± 3951.56 | 0.233 |
| Hex_6_(a) | 92274.93 ± 195728.70 | 11054.28 ± 9184.32 | 0.033 |
| Hex_6_(b) | 144891.81 ± 415107.36 | 45685.28 ± 149944.60 | 0.240 |
| Hex_6_(c) | 24872.05 ± 72765.02 | 63583.67 ± 286525.70 | 0.499 |
| Hex_7_(a) | 15148.75 ± 19160.46 | 62670.00 ± 138811.20 | 0.084 |
| Hex_7_(b) | 36273.00 ± 51680.28 | 57747.96 ± 131790.00 | 0.433 |
| Hex_7_(c) | 26393.65 ± 58767.75 | 29514.79 ± 67667.63 | 0.856 |
| Hex_7_(d) | 18414.00 ± 41139.36 | 10832.95 ± 13331.85 | 0.359 |
| Total | 1459983.07 ± 833705.59 | 821636.30 ± 751828.20 | 0.004 |

^1^Peak Area at 2 months, Ion Count and Peak Area at 5 months, Ion Count: Values are mean ± SD, *n* = 27 (2 months) or 28 (5 months) infants. EBF, exclusive breastfed; Hex, hexose; Non-EBF, non-exclusively breastfed; Pnt, pentose.

**Supplemental Table 7. Comparison of 2 months vs. 5 months of the absolute abundances of plant-based oligosaccharides for Non-EBF Infants^1^.**

| Oligosaccharide | Peak Area at 2 months, Ion Count | Peak Area at 5 months, Ion Count | Unpaired *t*-test *P*-value |
| --- | --- | --- | --- |
| Hex_3_(a) | 25364.23 ± 78975.04 | 119946.70 ± 416477.45 | 0.280 |
| Hex_3_(b) | 99514.77 ± 297937.20 | 103766.50 ± 263017.33 | 0.957 |
| Hex_3_(c) | 33302.86 ± 84729.49 | 56829.56 ± 129548.47 | 0.453 |
| Pnt_4_ | 44499.05 ± 39692.05 | 12988.48 ± 14638.93 | 0.011 |
| HexPnt_3_(a) | 25038.52 ± 50163.61 | 6771.78 ± 4756.93 | 0.065 |
| HexPnt_3_(b) | 10144.00 ± 18264.07 | 2442.04 ± 2004.64 | 0.034 |
| HexPnt_3_(c) | 33381.24 ± 31207.04 | 19677.78 ± 21599.33 | 0.072 |
| Hex_3_Pnt | 13784.29 ± 47920.20 | 1107.41 ± 1703.52 | 0.175 |
| Hex_4_(a) | 93559.58 ± 159151.30 | 283882.40 ± 557849.02 | 0.113 |
| Hex_4_(b) | 165324.80 ± 208991.20 | 389286.60 ± 1396330.46 | 0.441 |
| Hex_4_(c) | 59241.67 ± 58153.16 | 79335.89 ± 137742.55 | 0.510 |
| Hex_4_(d) | 141050.70 ± 224535.90 | 317267.60 ± 490291.15 | 0.113 |
| Pnt_5_(a) | 11294.33 ± 23492.49 | 6432.15 ± 9810.9611 | 0.330 |
| Pnt_5_(b) | 3841.43 ± 4252.41 | 6059.04 ± 13226.67 | 0.436 |
| Pnt_5_(c) | 12671.43 ± 40762.97 | 9327.70 ± 14318.33 | 0.691 |
| Hex_4_Pnt(a) | 26006.67 ± 40418.46 | 5993.00 ± 5856.34 | 0.014 |
| Hex_4_Pnt(b) | 21269.15 ± 47836.75 | 9367.41 ± 28101.00 | 0.278 |
| Pnt_6_(a) | 10064.50 ± 10464.04 | 6491.62 ± 6659.42 | 0.148 |
| Pnt_6_(b) | 8251.76 ± 11615.84 | 4075.24 ± 5464.40 | 0.101 |
| Hex_5_(a) | 42963.55 ± 68184.42 | 241622.60 ± 892993.18 | 0.283 |
| Hex_5_(b) | 29175.70 ± 22562.91 | 80720.48 ± 117979.69 | 0.041 |
| Hex_11_ | 47894.53 ± 86206.78 | 1157.83 ± 836.45 | 0.007 |
| Hex_12_(a) | 22120.00 ± 59201.85 | 5918.35 ± 10386.46 | 0.168 |
| Hex_12_(b) | 9259.50 ± 12112.50 | 2784.77 ± 2816.40 | 0.009 |
| Hex_6_(a) | 18158.30 ± 18273.22 | 121174.30 ± 262313.56 | 0.061 |
| Hex_6_(b) | 450037.70 ± 985453.30 | 45022.23 ± 138678.40 | 0.040 |
| Hex_6_(c) | 76277.95 ± 147465.70 | 36103.52 ± 118169.47 | 0.286 |
| Hex_7_(a) | 20805.11 ± 21117.79 | 73398.62 ± 94788.04 | 0.011 |
| Hex_7_(b) | 27928.21 ± 23350.78 | 48832.32 ± 83422.30 | 0.241 |
| Hex_7_(c) | 26400.95 ± 61879.01 | 42105.12 ± 123181.14 | 0.575 |
| Hex_7_(d) | 36299.11 ± 76519.61 | 9613.78 ± 11044.92 | 0.079 |
| Total | 1450651.92 ± 1080822.24 | 2046176.15 ± 3180505.45 | 0.387 |

^1^Peak Area at 2 months, Ion Count and Peak Area at 5 months, Ion Count: Values mean ± SD, *n* = 24 (2 months) or 27 (5 months) infants. EBF, exclusive breastfed; Hex, hexose; Non-EBF, non-exclusively breastfed; Pnt, pentose.

**Supplemental Figure 1.** Extracted ion chromatograms of an (A) EBF and (B) non-EBF infant. Peaks annotated without isomer letter designations were oligosaccharides not originally found in our in-house library. Data was analyzed using Agilent MassHunter Qualitative Analysis. Peak area data was used for statistics and receiver operating characteristic curves, extracted from Agilent MassHunter Quantitative Analysis. EBF, exclusive breastfed; Hex, hexose; Non-EBF, non-exclusively breastfed; Pnt, pentose.

**Supplemental Figure 2.** Peak area ion counts on plant-based oligosaccharides detected in EBF (black) and non-EBF (white) infant feces at 2 months (A) and 5 months (B). Values are means ± SDs, *n* = 27 (EBF) or 24 (non-EBF). EBF, exclusive breastfed; Hex, hexose; Non-EBF, non-exclusively breastfed; Pnt, pentose.

**Supplemental Figure 3.** ROC curves for fecal fructose (A) and mannose (B) measurements of EBF and non-EBF infants. Fructose was shown to have an AUC of 0.86 and mannose with an AUC of 0.82, indicative of good discrimination between EBF and non-EBF infants. For constructing the fructose ROC curve (A), *n* = 160 (EBF) or 28 (non-EBF). For the mannose ROC curve (B), *n* = 86 (EBF) or 98 (non-EBF). The dashed diagonal lines are the lines of identity representing an AUC of 0.50 (random chance). AUC, area under the receiver operating characteristic curve; EBF, exclusive breastfed; Hex, hexose; Non-EBF, non-exclusively breastfed; Pnt, pentose; ROC, receiver operating characteristic.
